# Chemical Compositions, Somatic Embryogenesis, and Somaclonal Variation in Cumin

**DOI:** 10.1155/2017/7283806

**Published:** 2017-11-06

**Authors:** Moslem Bahmankar, Seyed Mohammad Mahdi Mortazavian, Masoud Tohidfar, Seyed Ahmad Sadat Noori, Ali Izadi Darbandi, Giandomenico Corrado, Rosa Rao

**Affiliations:** ^1^Department of Agronomy and Plant Breeding, College of Aburaihan, University of Tehran, Tehran, Iran; ^2^Department of Plant Biotechnology, Faculty of Life Sciences and Biotechnology, Shahid Beheshti University, G.C., Tehran, Iran; ^3^Facoltà di Agraria, Università degli Studi di Napoli Federico II, Portici, Italy

## Abstract

This is the first report evaluating the relationship between the chemical compositions of cumin seeds (based on the analysis of the content of catalase, ascorbate peroxidase, proline, protein, terpenic compounds, alcohol/phenols, aldehydes, and epoxides) and the induction efficiency of somatic embryogenesis in two Iranian superior cumin landraces (Golestan and North Khorasan). Cotyledons isolated from Golestan landrace seeds cultivated on MS medium supplemented with 0.1 mg/L kinetin proved to be the best primary explant for the induction of somatic embryogenesis as well as the regeneration of the whole plantlet. Results indicated that different developmental stages of somatic embryos were simultaneously observed on a callus with embryogenic potential. The high content of catalase, ascorbate peroxidase, proline, and terpenic hydrocarbons and low content of alcoholic and phenolic compositions had a stimulatory effect on somatic embryogenesis. Band patterns of RAPD markers in regenerated plants were different from those of the mother plants. This may be related to somaclonal variations or pollination system of cumin. Generally, measurement of chemical compositions can be used as a marker for evaluating the occurrence of somatic embryogenesis in cumin. Also, somaclonal variations of regenerated plants can be applied by the plant breeders in breeding programs.

## 1. Introduction

Cumin (*Cuminum cyminum* L.) is one of the most important spicy and medical plants in the world, which belongs to the Apiaceae family. The Apiaceae family consists of 455 genera and over 3500 species and is regarded as one of the largest plant families [1, 2]. An effective and reproducible in vitro regeneration system is required for studies on genetic manipulation in each plant species [3]. Somatic embryogenesis is the developmental restructuring of somatic cells toward the embryogenic pathway and forms the basis of cellular totipotency in higher plants [4]. Somatic embryogenesis is one of the most effective techniques to be used in biotechnological studies such as producing transgenic plants, artificial seeds, micropropagation, and germplasm conservation [5, 6]. Somatic embryogenesis was initially reported in the Apiaceae family and in vitro culture of carrot [7, 8]. Genotypic properties of plants, physiological characteristics of the explant, and cell status in plant tissues can be mentioned as factors influencing somatic embryogenesis [9]. Somatic embryogenesis is a model system for understanding the physiological, biochemical, and molecular biological events occurring during plant embryo development [4]. Studies showed that various genotypes in a plant species have different embryogenesis capabilities, depending on genetic and biochemical properties, ability level of key elements in regeneration path, and also endogenous phytohormones metabolism [10]. Dave and Batra [11] conducted the first study of somatic embryogenesis in cumin using different explants including root, hypocotyls, and cotyledons. They stated that hypocotyl explants in MS medium complemented with 8 mg/L BA showed the best response to embryogenesis and emphasized the necessity of cytokinins hormone for production and development of cumin somatic embryos [11]. It seems that auxin hormones were stimulators of intracellular biochemical reactions and specific genes in preembryo cells, which are required for producing globular embryos. The researchers observed that elimination of auxin from medium can lead to the continuation of somatic embryogenesis [8, 12, 13]. Measurement of chemical compositions such as contents of proline, protein, and antioxidant enzymes including catalase, ascorbate peroxidase, and peroxidase has been used as a marker to evaluate somatic embryogenesis in different studies [10, 14, 15]. Studies suggest that antioxidant enzymes such as catalase and ascorbate peroxidase play an important role in maturity and differentiation of plant tissues and their high activity affects cellular processes such as somatic embryogenesis [10, 16, 17]. Terpenic hydrocarbons, alcohols, and phenols existing in the essential oil of plants are among the genetic properties affecting somatic embryogenesis. Researchers stated that terpenic hydrocarbons have positive effects on somatic embryogenesis process, while alcohols and phenols have negative effects on the process [18–20]. Ebrahimie et al. [3] used the cut seed embryo of cumin as an explant in B5 medium and introduced 0.4 mg IAA + 0.2 mg NAA + 0.1 mg BAP as the best hormone combination. Kahrizi and Soorni [21] used 2,4-D and Kin in cumin developed embryogenic calluses and reported that an increase in the concentration of 2,4-D hormones has a positive effect on somatic embryogenesis, but their study was only confined to callus formation and they did not report on regeneration and adaptation. Although few studies were conducted on somatic embryogenesis and regeneration in cumin, already no report has been registered on the relationships between genetic properties like chemical compositions existing in the plant and somatic embryogenesis process and evaluation of somaclonal variation of regenerated plants. Therefore, the current study is aimed at measuring chemical compositions, optimizing somatic embryogenesis and regeneration, assessment of somaclonal variation in regenerated plants as compared to the mother plant, and then evaluating relationships of these properties in the two superior Iranian landraces of cumin.

## 2. Materials and Methods 

### 2.1. Plant Material

**Figure 1:**
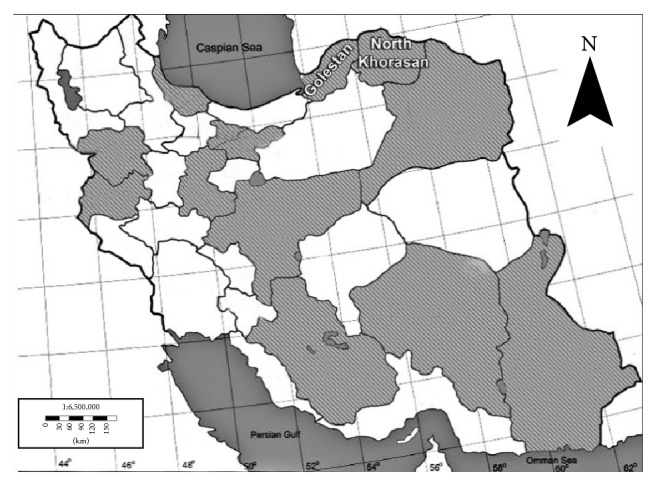
Location of two superior Iranian cumin landraces on the map (modified from http://www.enchantedlearning.com).

Two Iranian superior cumin landraces seeds, Golestan and North Khorasan, were selected based on the previous study [22] (Table 1 and Figure 1). In the present study, experiments were divided into two sets including measurement of chemical characters and optimization of somatic embryogenesis, regeneration, rooting, and evaluation of somaclonal variation of the landraces of cumin.

### 2.2. Measurement of Chemical Characters

In the first experiment, the seeds of two landraces of cumin were planted in the experiment site of College of Aburaihan, University of Tehran, during two successive years (2014 and 2015). Each landrace was planted in 2 m^2^ plots in a sandy-clay soil. The soil was well drained, with pH of 7.2. Recommended crop management practices were implemented to raise the crops to desirable growth stages. Plots were kept weed-, pest-, and disease-free until harvesting time. Chemical characters including the content of catalase, ascorbate peroxidase, protein, proline, essential oil, and its composition of two landraces of cumin were measured. It is worth noting that catalase and ascorbate peroxidase play an important role in the maturity of plant tissues and their high activities affect somatic embryogenesis [10, 16, 17]. Each essential oil is made up of numerous different organic molecules and some of these including terpenic hydrocarbons, alcohols, and phenols existing in the essential oil are among the genetic properties affecting somatic embryogenesis [18–20]. In order to measure chemical characteristics of two landraces of cumin, leaf tissue samples were immediately frozen in liquid nitrogen. Protein and proline contents of the leaves were measured according to Bradford and Bates et al. [23, 24], respectively. The activities of antioxidant enzymes including catalase and ascorbate peroxidase were estimated as described by Beers and Sizer [25] and Nakano and Asada [26]. The essential oil of the ripened seeds was extracted by the Clevenger apparatus, using the hydrodistillation method. The dried powdered seeds of cumin (40 g) were placed in a distillation apparatus with 400 mL of distilled water and hydrodistilled for three hours. Then, the essential oils were stored in glass vials at 5°C until the essential oil compositions' analysis. The analysis of essential oil composition was performed by GC-MS analysis (a combined analytical method to identify different substances within a sample): Varian CP-3800 GC (gas chromatography) coupled with Varian 4000 (ion trap) MS (mass spectrometry) equipped with a capillary VF-5 fused silica column (30 m × 0.25 mm i.d., film thickness: 0.25 *μ*m). Helium was used as the carrier gas at the constant flow of 1.0 ml min^−1^, with split ratio of 1/50. Mass spectra were taken at 70 Ev and Mass range was from *m*/*z* 35–400 a.m.u. The oven temperature was held at 60°C for 1 min and then programmed to 250°C at a rate of 3°C min^−1^ and held for 10 min. The injector and detector (FID) temperatures were kept at 250 and 280°C, respectively. The essential oil compositions were identified by calculation of their retention indices under temperature-programmed conditions for n-alkanes (C6–C24) and the oil on a VF-5 column under the same chromatographic conditions. The compounds were identified by comparison of their mass spectra with those of the internal reference mass spectra library (Wiley 7) or with authentic compounds and confirmed by comparison of their retention indices with authentic compounds or with those reported in the literature. For quantification purposes, relative area percentages obtained by FID were used without the use of correction factors.

#### 2.2.1. Experimental Design and Data Analysis

A randomized complete block design with five replications was used. Seeds were sown by hand with 10 cm distance in rows. The data related to chemical characteristics were recorded during two years (2014 and 2015) and means of these were used for analysis. Five samples were recorded in each plot. Data for the experiment were subjected to paired samples *T*-test to evaluate the statistical significance and were expressed as the mean ± SE. SPSS 16.0 and Excel software were used for data analysis.

### 2.3. Optimization of Somatic Embryogenesis and Regeneration

In second set of experiment, the cumin seeds were surface-sterilized following the procedure previously described [22]. To obtain plantlets, the sterilized seeds were cultured in the half-strength MS medium and stored in the growth chamber [21]. One month after seed culturing, when grown plantlets were about 5 cm in height, about 0.5 cm of each explant including hypocotyl and cotyledon was prepared. The prepared explants were cultured in MS medium containing 3% (w/v) sucrose and 0.8% (w/v) agar and supplemented with 7 different concentrations of PGRs (2,4-D and Kin) (Table 2). The cultured explants were incubated in the growth chamber at 24°C ± 2°C, 75% relative humidity, and dark condition during the first month of culture. After this step, the formed calli were subcultured into fresh mediums containing the previous combination of PGRs and then were stored in the growth chamber at 24°C ± 2°C and 75% relative humidity under 16/8 h photoperiod with 50 *μ*mol^−2^ s^−1^ photosynthetic photon flux density provided by cool white fluorescent light. Data related to the percentage of callus formation was recorded five weeks after culture. Seven weeks after culture, calli images were taken by a camera under the same situations and then perimeter and area of calli were calculated by Digimizer, an image analysis software (version 4.3.1, MedCalc Software, Belgium). In the eighth week, the symptoms of somatic embryogenesis in some of the studied mediums were observed without transferring the calli to the PGRs-free medium; then different stages of the somatic embryogenesis were recorded using a microscope.

Thereafter, the calli were transferred to the PGR-free medium for excluding of auxin effect and further stimulation of somatic embryogenesis. The percentage of induced calli with embryogenic potential was calculated on the basis of a number of explants producing somatic embryogenesis/number of total cultured explants in each Petri dish after two to three weeks. Data related to the percentage of plant regeneration were recorded during 12 weeks after the first culture. The percentage of plant regeneration was calculated on the basis of the number of regenerated explants/the number of induced calli with embryogenic potential in each Petri dish.

#### 2.3.1. Experimental Design and Data Analysis

The experiment was performed in a factorial form using a completely randomized design with three factors and three replications (as Petri dishes). Five explant segments were placed in each Petri dish containing 25 ml different medium. Studied factors included two Iranian cumin landraces, Golestan and North Khorasan, two explants, hypocotyl and cotyledon, and 7 different concentrations of PGRs (Table 2). The data related to the percentage of callus formation, perimeter, and area of calli were subjected to ANOVA and DMRT to evaluate the statistical significance and were expressed as the mean ± SE. Since the distribution of the recorded data related to measured characters including the percentage of induced calli with embryogenic potential and the percentage of plant regeneration was not normal and no transformation was able to induce normality, the Kruskal-Wallis and the Mann–Whitney nonparametric tests were used and median grouping was made [27]. SAS 9.2, SPSS 16.0, and Excel software were used for data analysis.

### 2.4. In Vitro Rooting and Acclimatization

Since the best response of plant regeneration was observed in the MS medium containing 0.1 mg/L Kin, the regenerated 3 to 4 cm long plantlets were excised from mentioned medium and then were cultured in pots containing half-strength MS medium. The following treatments were investigated for more rooting: MS + PGR-free, MS + IAA (0.35 mg/L), MS + NAA (0.37 mg/L), and MS + IAA (0.35 mg/L) + NAA (0.37 mg/L). Pots were observed every 3-4 days regularly and then some of the characters including days to production of the main rooting, the percentage of produced main rooting, and the percentage of produced lateral rooting were recorded to compare investigated treatments. After 3 weeks, sufficiently developed cumin plantlets containing rooting were transplanted into plastic pots containing sterilized garden soil. Then, the pots were covered with the plastic cover for maintaining of plants moisture and kept in the greenhouse for two weeks. Thereafter, they were acclimatized to greenhouse conditions by removing the covering plastic. Percentage of survival plantlets was recorded and then they were kept until flowering and seed production.

#### 2.4.1. Experimental Design and Data Analysis

The rooting experiment was performed as a completely randomized design with three replications (as pots). Ten regenerated plantlets were placed in each pot containing 50 ml of investigated medium. The data related to the rooting experiment were subjected to ANOVA and DMRT to evaluate the statistical significance and were expressed as the mean ± SE.

### 2.5. Evaluation of Somaclonal Variation by RAPD Markers

Somaclonal variation is the variation seen in plants that have been produced by plant tissue culture. It is particularly common in plants regenerated from callus [28, 29]. Typical somaclonal variations are the change in chromosome number, chromosome structure, and DNA sequence [29]. In this experiment, genetic homogeneity of the regenerated plantlet as compared to mother plants was examined by RAPD markers. Total genomic DNA was extracted from young leaves of the in vitro regenerated cumin and mother plant following the CTAB procedure according to Murray and Thompson [30] with minor modifications. Quality and quantity of extracted genomic DNA were checked on 1% agarose gel and spectrophotometer and the samples were diluted to a final concentration of 50 ng/*μ*l. Ten RAPD primers (OPU6, OPU12, OPS17, OPJ18, OPJ19, OPJ21, OPC13, OPC8, OPQ6, and OPF5) were used for DNA amplification [31]. PCR amplification was performed in a 25 *μ*l reaction volume containing 9.5 *μ*l double-distilled water, 12.5 *μ*l PCR amplification kit (10x), 1 *μ*l primer (10 p mol), and 2 *μ*l DNA (50 ng/*μ*l). The amplification program included initial 5 min denaturation at 95°C, followed by 30 cycles of 1 min at 95°C, 30 sec at 35°C, and 2 min at 72°C, with a final extension at 72°C for 8 min. The PCR products were electrophoresed on 2% agarose gels, stained with ethidium bromide, and visualized on a UV light transilluminator.

## 3. Results and Discussion

### 3.1. Chemical Characters

**Figure 2:**
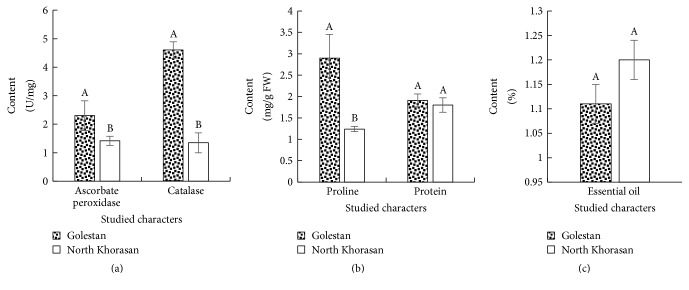
Means comparison of studied chemical characters ((a), (b), and (c)) in the studied cumin landraces based on paired-samples *T*-test. In each character, landraces with one common letter at least are not significantly different at the 5% level.

The antioxidant enzymes play a role in metabolizing reactive oxygen species and preventing damage due to oxidative stresses, auxin metabolism, cross-links in the cell wall, and response to environmental stresses [17, 32]. Results of the present study showed that level of catalase and ascorbate peroxidase enzymes in Golestan landrace was significantly much more than that in North Khorasan landrace (Figure 2(a)). The presence of catalase in plant cells plays an important role in increasing resistance to oxidative stresses. In addition to ascorbate peroxidase catalyzing the H_2_O_2_ reduction reaction, also this enzyme plays a role in many cellular processes such as auxin metabolism, cross-links in cellular wall, and response to environmental stresses [10, 16, 32].

The results suggested that, in terms of protein contents, there was no significant difference between the two landraces of Golestan and North Khorasan (Figure 2(b)). The protein content of cells is regarded as one of the most important factors of cell growth [33]. Proteins play important physiological roles such as structural and enzymatic roles on the level of cells and it is not possible for cells to continue growing in the long run without these materials. Any factors that inhibit protein synthesis lead to cell growth failure [32, 34]. Proline is also the key amino acid that develops stress tolerance in plants through protection and sustainability of cell membranes [32, 34]. The results showed that Golestan landrace had higher proline content than North Khorasan landrace (Figure 2(b)). Proline in addition to its osmotic role is effective as a source of nitrogen for rapid growth of plant cells [33, 35]. There was no significant difference between the two landraces in terms of the amount of essential oil (Figure 2(c)). In terms of chemical structure, the essential oils are not homogeneous and have different compositions. Dubey et al. [36] reported significant differences between the Indian landraces of cumin in terms of the amount of essential oil.  Table 3 represents GC-MS profile of essential oil of two studied cumin landraces. The results showed that the identified compounds in their essential oil may be classified into five organic groups including terpenic hydrocarbons, alcohols and phenols, aldehydes, epoxides, and other compositions (Table 3).

It should be mentioned that the two groups of terpenic hydrocarbons and aldehydes were the highest existing compositions in the essential oil of the studied cumin. In a study on the chemical compositions of the landraces of Indian cumin, the main ingredients of essential oil of terpenic hydrocarbons and aldehydes were reported [2]. In the present study, the extracted essential oil from Golestan landrace contained 62.27% terpenic hydrocarbons, while the amount of these compositions was 51.14% in North Khorasan landrace (Table 3). It would be worth noting that two compositions of alpha-thujene and gamma-terpinene were the main constituent elements of terpenic hydrocarbons and their expanding range varied from 6.91% to 24.8%. Alpha-thujene was significantly higher in North Khorasan landrace, while the number and amount of other identified terpenic hydrocarbons in Golestan landrace were higher compared to North Khorasan landrace (Table 3). In the present study, a total of 45 compositions were identified by GC-MS based analysis of cumin essential oil, representing 98.42 to 99.15% of the total compositions (Table 3). In other studies, some researchers reported that the total numbers of identified compositions in Egyptian landraces, Indian landraces, Chinese landraces, and Tunisian landraces were 41, 29, 19, and 38, respectively [2]. Discrepancies in the results reported on chemical compositions of cumin essential oil may be due to factors including the sensitivity of the system used or method adapted for analysis. The chemical compositions of the essential oil of medicinal plants are affected by numerous internal and external factors including plant genotypes, geographical location, climatic conditions, growth season, and essential oil extraction method [2, 37]. Analysis of essential oils of the two landraces showed that, among the identified compositions in the essential oils, cumin aldehyde has been the main constituent of cumin essential oil whose value in the Northern landrace has been significantly higher compared to Golestan landrace (Table 3). Cumin aldehyde smells good and is the main reason for the aromatic smell of cumin seeds and spice. This substance is commercially used in perfume and in hygienic and cosmetic industries. It is worth noting that, in local and international markets, cumin seeds containing more cuminaldehyde are preferred to cumin seeds containing terpinene [2].

### 3.2. Somatic Embryogenesis and Regeneration

**Figure 3:**
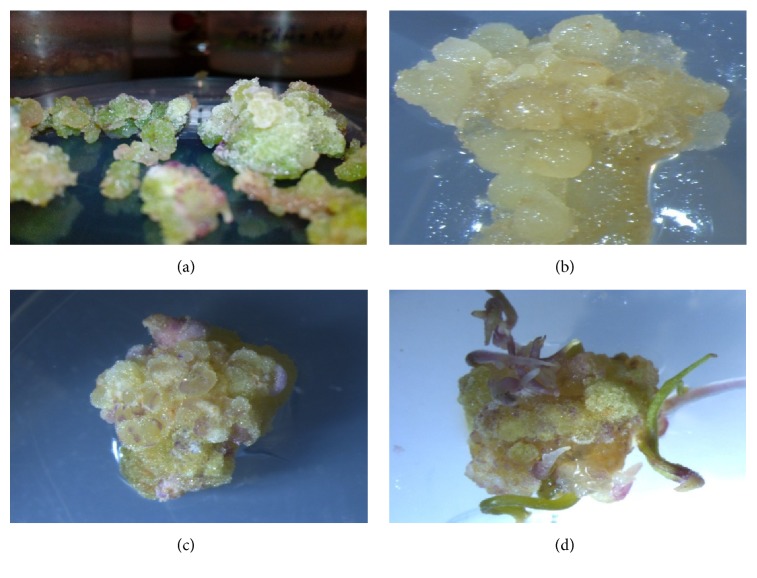
Nonembryogenic ((a) and (b)) and embryogenic (c & d) calli in cumin.

Two types of embryogenic and nonembryogenic calluses were observed in different treatment combination (Figure 3).

**Figure 4:**
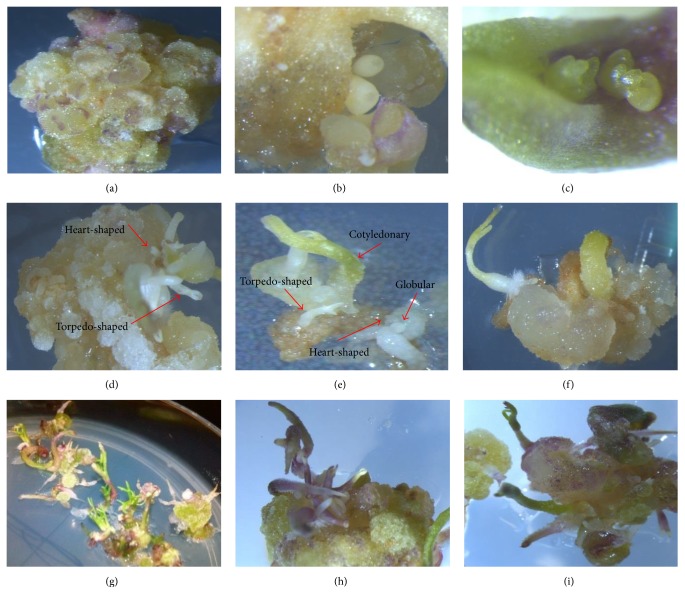
Different developmental stages of somatic embryogenesis and plant regeneration in cotyledon of cumin (*Cuminum cyminum* L.) Golestan landrace onto MS medium containing 0.1 mg/L Kin. ((a), (b), and (c)) Globular embryos developing from cotyledon explants. ((d), (e), and (f)) Simultaneous induction stages of the somatic embryo from cotyledon explants. ((g), (h), and (i)) Regenerated and elongated shoots from induced somatic embryo.

The knotted and compact calluses were embryogenic calluses. Nonembryogenic calluses were agglomerate and fragile and their size was much larger than embryogenic calluses. Somatic embryos were observed in the outer layer of calluses about 2-3 weeks after the transfer of calluses to embryogenesis induction medium (PGRs-free). It is worth noting that different developmental stages of somatic embryos (from globular to cotyledonary stage) were simultaneously observed on a callus with embryogenic potential (Figure 4).

This observation suggests that development of somatic embryos is highly unsynchronized process in cumin. The long-term culture of embryo increased the number of embryos, but all embryogenic callus sections were not able to produce globular embryos. It is worth noting that researchers found that the embryogenic ability was not the same in calluses; only the globular calluses with soft surfaces had the embryogenic ability and rough, dry, and brittle calluses did not have embryogenic ability [38, 39]. Some researchers reported that differentiation of somatic embryos in cumin was observed about two weeks after calluses transfer to PGRs-free MS medium [21]. Results of another study suggested that somatic embryos appeared one week after the calluses transfer to a PGRs-free medium [8]. The results of the present study indicated that, among the studied factors and their interactional effects on the percentage of callus formation, only explants effect was significant, so that cotyledon explant showed a superior response compared to hypocotyl explant (Table 4).

**Figure 5:**
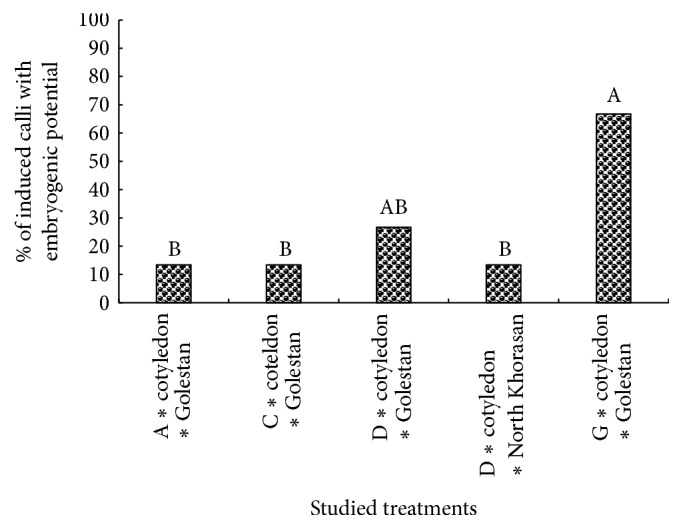
Rank comparisons of percentage of induced calli with embryogenic potential in the studied treatments. Values in each column followed by the same letters are not significantly different according to the Kruskal-Wallis nonparametric test.

**Figure 6:**
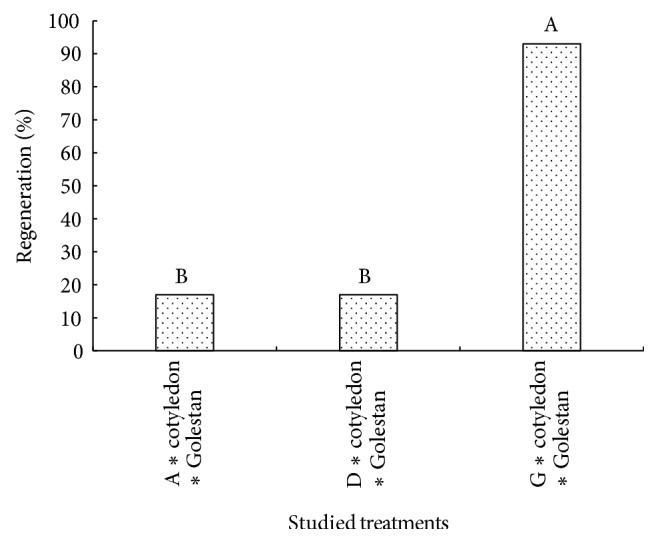
Rank comparisons of plant regeneration% in the studied treatments. Values in each column followed by the same letters are not significantly different according to the Kruskal-Wallis nonparametric test.

The sensitivity of cultured cells and tissues to external hormones signaling can determine their embryogenic ability. Some researchers stated that somatic tissues of plants varied in terms of callus production and also cotyledon embryogenesis sensitivity was higher than hypocotyls [40, 41]. Results showed that the interactional effect of medium, landrace, and explant on the percentage of callus formation was not significant, while these significantly influenced callus perimeter and area (Table 4). It is worth noting that most perimeter and area of callus were observed in hypocotyl explant of North Khorasan on 0.5 mg/L 2,4-D + 0.1 mg/L Kin medium (Table 4). In order to study the effect of hypocotyl length and placement in the medium on callus properties such as callus perimeter, area, and density, Mansouri et al. [42] stated that the main and interactional effects of intended factors had no significant effect on the callus properties. In the present study, the overall assessments suggested that most callus dimensions (callus perimeter and area) have been obtained in 0. 5 mg/L 2,4-D + 0.1 mg/L Kin growth medium and also hypocotyl explant was superior in terms of the callus properties (Table 4). Since no induced calli with embryogenic potential and regeneration were observed in most treatment combinations, their analysis was excluded. Due to the fact that the data associated with the percentage of induced calli with embryogenic potential and regeneration percentages were not normally distributed and were not made normally distributed through data transformation methods, Kruskal-Wallis nonparametric method was used to analyze these. The results showed that the highest percentage of induced calli with embryogenic potential and regeneration was achieved in treatment combination of cotyledon explant of Golestan landrace in a medium containing 0.1 mg/L Kin (Figures 4, 5, and 6). In this study, it was observed that cotyledon was superior to hypocotyl in terms of induced calli with embryogenic potential and regeneration and hypocotyl showed no somatic embryogenesis response.

It should be noted that the level of endogenous hormones is one of the required factors affecting the responding ability of explants. Quantity and quality of endogenous hormones vary across explants, so they affect their responding potentiality [43, 44]. Some researchers believe that although the totipotency property is a very important trait in plants, cells only gain this ability under certain conditions. Even when it is thought that all cells are equally totipotent, the number of embryos as compared to the number of cells in cellular mass is insignificant. Thus, only a limited portion of the cell mass has the ability to produce somatic embryos, and this process is confined to the particular tissue of a genotype [4, 39, 45]. Experiences gained through tissue culture indicate that there is a type of slope between different parts of the plant in response to embryogenesis, so that embryogenic ability has been high in embryo-derived tissues and low in hypocotyl and root [41]. Results of the present study suggest that somatic embryogenesis is highly dependent on the type of explant, genotype, and culture medium. Other researchers have also reported that the type and concentration of growth regulators and physiological state of explants are the most important factors, which play a role in somatic embryogenesis [8, 39]. In the present study, the level of percentage of induced calli with embryogenic potential and regeneration under the treatment of 0.1 mg/L Kin was significantly higher than other treatments, which suggest the role of cytokinin hormones in somatic embryogenesis (Figures 5 and 6).

It is worth noting that, in the first embryogenesis experiment conducted on cumin, the role of cytokinin hormones for the occurrence of this process has been emphasized [11]. According to the results of the present study, although induced calli with embryogenic potential have been observed in other media containing hormone combination of auxin and cytokinin (0.5 mg/L 2,4-D + 0.1 mg/L Kin) or auxin alone (0.5 mg/L 2,4-D), the rate of regeneration in these was significantly lower than that in the medium containing cytokinin (Figures 5 and 6). It would be worth noting that induced calli with embryogenic potential were also observed in some treatment combinations containing 2,4-D in the present study (Figure 5). This process can be attributed to the key role of auxin in the induction of somatic embryogenesis which can directly or indirectly regulate embryogenesis process through affecting the internal metabolism of other phytohormones [39, 46]. It should be mentioned that, in the presence of 2,4-D, embryos were not able to pass through the globular stage to heart-shaped stage and then complete embryogenesis. The inhibitory effect of 2,4-D hormone was also reported in other plants belonging to Apiaceae family including carrot and celery [47]. It is worth noting that percentage of induced calli with embryogenic potential and the rate of regeneration have decreased in treatment combinations containing higher auxin concentration compared to treatment combinations containing lower auxin concentration (Figures 5 and 6). Higher concentration of auxin hormones in the culture medium resulted in the biosynthesis of hydrocarbons such as ethylene, which results in cells and plant organs getting old. In fact, auxin acts as an effective inducer of somatic embryogenesis whose reduction and omission from the culture medium resulted in developing somatic embryogenesis [48]. Therefore, researchers have recommended prevention from continued exposure to high levels of auxin during the somatic embryogenesis process [8, 13]. Some researchers also believe that sometimes auxin in the culture medium is decomposed through photooxidation and leads to undesirable effects on the effectiveness of somatic embryogenesis through the production of unwanted compositions [49, 50]. It should be noted that plants in Apiaceae family such as cumin contain high levels of secondary metabolites and endogenous growth regulators, particularly auxin and cytokinins. Therefore, it is possible that high levels of endogenous regulators in the plant have caused the external use of them to be unnecessary [51]. Valizadeh et al. [52] have mentioned the occurrence of regeneration in some kinetin-free treatments as reasons for this claim. The researchers believe that the most important factor involved in differences between embryogenic and nonembryogenic cultures is their difference in terms of the level of endogenous hormones because these regulate explants differentiation processes in the media [38, 53]. In general, factors such as plant genetic properties, type of explants, the explant growth phase, and cells position in plant cultured tissue are effective for their somatic embryogenic ability.

### 3.3. In Vitro Rooting and Acclimatization

**Figure 7:**
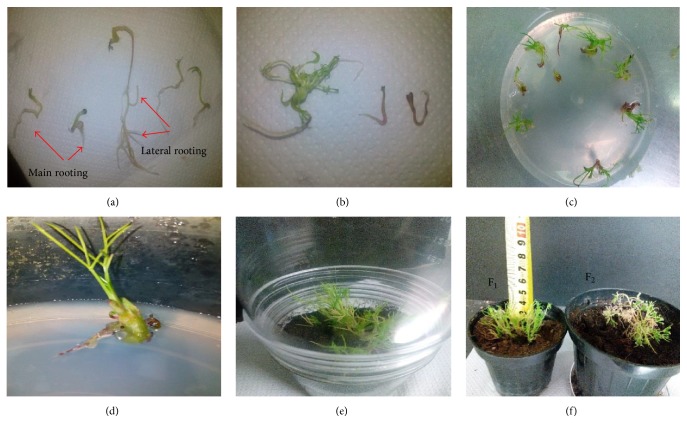
Induction of rooting and developed plantlets in cumin (*Cuminum cyminum* L.) Golestan landrace. ((a) and (b)) Main and lateral rooting in MS medium containing IAA (0.35 mg/L) + NAA (0.37 mg/L). ((c) and (d)) Regenerated cumin planted. (e) Pot covered by a plastic cover. (F1) Developed cumin plantlets and (F2) mature cumin plant.

The results showed that the hormone treatment IAA (0.35 mg/L) + NAA (0.37 mg/L), as compared to other treatments, was superior in terms of rooting rate, the percentage of lateral root production, and survival rate of regenerated seedlings (Figure 7). According to these results, it can be said that IAA and NAA auxin have synergistic effects on properties related to rooting and act supplementally (Table 5). Ginger also was observed among medical plants, regenerated organs of which have produced roots under the application of IAA and NAA hormones [54]. Rooting in plants is usually induced in media containing auxin hormones [54]. In the present study, in the medium free of auxin, limited rooting was also observed which may be associated with plant genotypes and endogenous hormone levels (Table 5). It is worth noting that some researchers also reported that cumin regenerated seedlings have produced roots in the auxin-free medium, but roots produced in the growth medium containing IBA auxin were much more and stronger [55]. Other researchers reported that rooting in calluses obtained from hypocotyl of cumin has been significantly increased with an increase in concentration of NAA hormone [52]. Researchers found that the usage of IBA hormone to induce rooting in cumin is effective and reported that although the usage of PEG in the medium has led to decreased rooting in cumin, it has increased survival ability of regenerated plants [55]. The researchers believe that PEG has induced morphological and anatomical changes associated with increased viability and adaptability of plants [56, 57]. Since the survival rate of plants regenerated in the present study was lower than the rate observed by Tawfic and Noga [55], perhaps in addition to differences in the type of genotype, lack of PEG can be mentioned as another reason for it. One of the most important problems of micropropagation is the survival rate of the seedling during transferring to ex vitro conditions to acclimatization to the new climate in the greenhouse or field. The type of environment and pot soil are also factors affecting the establishment and the survival rate of regenerated seedlings [55].

Generally, decreased survival rate of plants regenerated in tissue culture experiments can be attributed to different factors including low photosynthetic capacity resulting from the presence of sugar in the culture medium, low light, inadequate amount of CO_2_, and high relative humidity in the medium, which eventually undesirably affect the efficiency of the plant's photosynthesis and its survival rate [58].

### 3.4. Evaluation of the Relationship between Chemical Composition and Somatic Embryogenesis

The processes of cell differentiation and somatic embryogenesis are highly dependent on the genetic and chemical composition of plants. Measurement of chemical parameters can be used as a marker to assess somatic embryogenesis [14, 15]. Studies suggest that antioxidant enzymes such as catalase and ascorbate peroxidase play an important role in the growth and differentiation of plant cells and tissues and their high activities are associated with cell differentiation process [10, 16, 17]. The results of the present study showed that the contents of catalase and ascorbate peroxidase and the percentage of induced calli with embryogenic potential in Golestan landrace were significantly higher compared to North Khorasan landrace. Since somatic embryogenesis is heavily dependent on the genotype, one of the factors which are effective in somatic embryogenesis differences among the studied cumin landraces may be significant differences in their chemical compositions [5, 59, 60]. Studies have shown that ascorbate peroxidase is required to maintain size and shape of cells during embryogenesis. It also plays an important role in the formation of lignin and protects embryo cell walls [10, 61]. Researchers have also stated that variations in the content of antioxidant enzymes correlate with the regeneration of plants [10, 16, 32]. It is worth noting that catalase and ascorbate peroxidase have been used as markers to study the developmental stages of somatic embryos and their highest activity level was observed in torpedo stage of the somatic embryogenesis [10]. In the present study, Golestan landrace, in addition to antioxidant enzymes, had much more proline and protein. These compositions may be considered as other genetic properties of this landrace which lead to increased somatic embryogenesis in it. It should be mentioned that increased somatic embryogenesis through proteins and amino acids is due to the fact that the compositions easily supply the source of nitrogen for the cell growth [35]. Proline plays an important role in somatic embryogenesis and stress conditions in plants [62]. For example, studies have shown that the proline-rich protein is required in the cell wall of* Arabidopsis* for correctly placing cell plate during cytokinesis process for embryos development. The carrot cell wall protein has been secreted into the growth medium and regulates somatic embryogenesis development [63]. Researchers observed that protein content of cells in preembryogenesis calluses was higher than that in embryogenic calluses. In fact, as the callus enters somatic embryogenesis stage, its protein level decreases, which indicates protein consumption for somatic embryogenesis development. Studies have shown that cells use their protein content to initiate embryogenesis, so it can be said that the cellular protein content is among genetic properties that lead to increased somatic embryogenesis [10, 34]. As somatic embryogenesis initiates and develops, an increase in protein consumption has also been reported in other studies [9, 10]. The cultured cells are naturally able to synthesize all their necessary amino acids; however, embryogenesis and regeneration levels in cumin can be increased by adding proline and other amino acids to the culture medium, and this ability can be applied in gene transfer programs [35, 59]. Results of the essential oil analysis showed that phytochemical profiles of the two landraces of Golestan and North Khorasan have significant differences with each other, which is another confirmation of the existence of genetic differences between them. For example, the number of terpenic hydrocarbons was higher in essential oil of Golestan landrace than that in North Khorasan landrace (Table 3). Researchers have reported that the genetic characteristics are regarded as terpenic hydrocarbons, which could have a positive effect on somatic embryogenesis in plants [20]. The results of this study suggest that one of the main factors affecting the occurrence of somatic embryogenesis in Golestan landrace is the phytochemical properties, especially the high terpenic hydrocarbons content. It should be noted that North Khorasan landrace has higher alcoholic and phenolic compositions and these compositions along with other factors may lead to decreased somatic embryogenesis in it. How phenolic compositions affect the induction and development of somatic embryo is not clear yet, but these compositions may be involved in the metabolism of endogenous hormones such as auxin [18, 19]. Phenolic compositions prevent the growth and development of somatic embryogenesis from going beyond the globular stages. Embryos formed in the presence of phenolic compositions lack sufficient amounts of sucrose to carry out the metabolic activity and thus stop in the globular stage. It should be noted that the inhibitory effect of phenolic compositions due to their high concentration in cells leads to protein deposition, enzyme deactivation, or membrane damage [18, 64]. However, in some studies, it has been reported that phenols usually have a reinforcing effect on somatic embryogenesis, but it is possible that phenols interaction with alcohols and other phytochemical compositions of essential oil inhibits their reinforcing effect on embryogenesis. Results of the present study showed that essential oil percentage of North Khorasan was higher than Golestan landrace; however, the difference was not significant. Also, it should be noted that cuminaldehyde content, the most primarily essential oil composition of cumin, was significantly higher in North Khorasan landrace than in Golestan landrace. A high percentage of essential oil and more cuminaldehyde content in North Khorasan landrace may be regarded as two of the factors affecting the lack of its somatic embryogenesis. As no study has been reported on the effect of cuminaldehyde on somatic embryogenesis in cumin, the final decision on this issue requires further studies. Results of the present study showed that variation in the genetic and chemical structure of cumin landraces is among the important factors determining somatic embryogenesis process and can be used as a marker for assessment of occurrence of this process.

### 3.5. Evaluation of Somaclonal Variation in the Regenerated Plants in Comparison with the Mother Plants

**Figure 8:**
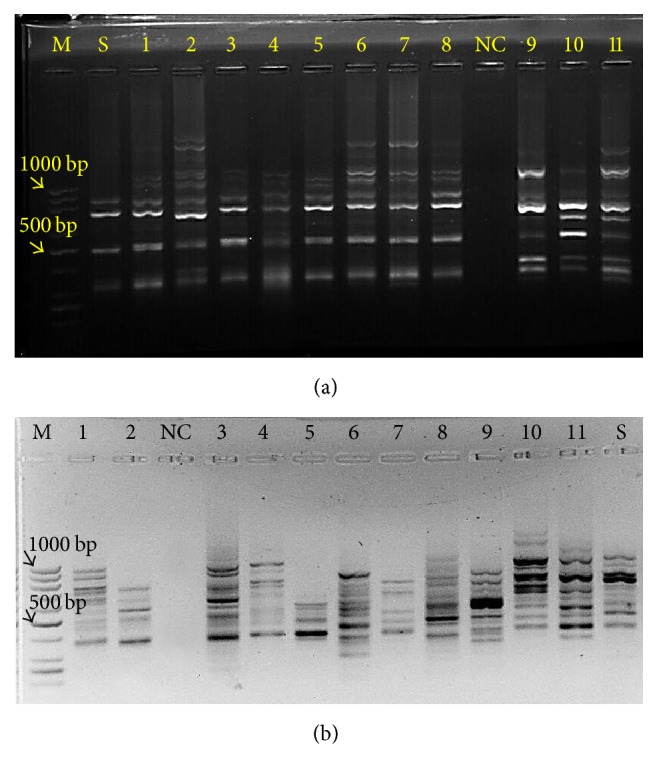
RAPD marker analysis generated by the primers OPC8 (a) and OPU12 (b). Lane M corresponds to 100 bp DNA ladder. Lanes 1–11 correspond to DNA from randomly selected regenerated plantlets. Lane S corresponds to seed plantlet. Lane NC corresponds to negative control.

Somaclonal variation is a common process in plants cell and tissue culture and was firstly introduced by Larkin and Scowcroft [28]. This process has led to changes in nuclear and cytoplasmic genomes and is of genetic and epigenetic nature [28, 29]. Ten primers were initially used, 7 of which generated informative and reproducible bands (6–10 bands per primer), ranging from approximately 200 to more than 1000 bp in size. The 7 primers generated a total of 52 scorable bands, with an average of 7.4 per primer, which were polymorphic across all the analyzed in vitro regenerated plantlets originating from cotyledons of the mother plant (Figure 8). In the present study, RAPD markers suggested polymorphism bands between regenerated plants of Golestan landrace and seed plants (Figure 8). It may be said that the variation is associated with the heterozygous nature of the cumin landrace and its pollination system. Some studies suggest somatic variations in plants regenerated from tissue culture and some others suggest genetic stability [65–67]. Bakhtiar et al. [67], in their study on the genetic diversity of* Thymus* regenerated seedlings and seedlings from mother plant, found that RAPD marker suggests the presence of polymorphism bands among them.

They believed that the observed variation may be related to* Thyme* pollination system. They also stated that the optimized protocol for* Thyme* regeneration is a useful tool to protect germplasm and it does not seem that interferences have been made in the genetic uniformity of the regenerated plants [67]. Analysis of* Arabidopsis* regenerated using RAPD and RFLP markers indicated the lack of diversity in the DNA profile of regenerated plants [68]. Evaluation of somatic variation frequency is valuable for any regeneration system, but the variation has been studied in few regenerated plants [43]. Although molecular markers have been used as a molecular tool to evaluate somaclonal variation, with regard to the fact that morphological and cytogenetic diversity cannot be detected at DNA level, there is a little doubt about their effectiveness [29]. In regenerated plants, changes in DNA methylation levels are considered as another source of somaclonal variation. It is believed that the occurrence of somaclonal variation resulting from DNA methylation, especially when auxin is consumed, is more than somatic variation due to changes in the DNA sequence [69, 70]. It is also well known that increasing numbers of subcultures increases the likelihood of somaclonal variation [70]. Genetic uniformity of produced plants through tissue culture depends on several factors; the most important ones are the propagation method and genotype [51, 67]. As compared to classical methods of vegetative propagation, the regenerated plants from calluses, depending on the species, origin, and culture age, may show varying amounts of structural or physiological disorders. Although these disorders seem inappropriate at first, since a limited number of the above-mentioned plants have desirable characteristics missing in the main plants, breeders will use this genetic variation in breeding programs [51].

## 4. Conclusion

Results of this study identified effective factors in somatic embryogenic ability such as plant genetic properties, type of explant, and type and concentration of PGRs and the cotyledon of Golestan landrace in MS + 0.1 mg/L Kin medium was the best treatment. The results also suggested that different developmental stages of somatic embryos were simultaneously observed on a callus with embryogenic potential. This observation suggests that development of somatic embryos is a highly unsynchronized process in cumin. The high content of catalase, ascorbate peroxidase, proline, and terpenic hydrocarbons and low contents of alcoholic and phenolic compositions had a positive effect on somatic embryogenesis. Therefore, measurement of these characters may be useful as a marker to predict somatic embryogenesis in cumin. Results suggested that although the cultured cells are naturally able to synthesize all their necessary chemical parameters, modulation of them through manipulation of culture medium may be an effective method to improve somatic embryogenesis in cumin and it can be applied in gene transformation. Band patterns of RAPD markers in regenerated plants were different from those of the seed plants. It may be related to somaclonal variations or impure nature and pollination system of cumin. It should be noted that plant breeders can apply the observed variation of regenerated plants from tissue culture in breeding programs.

## Figures and Tables

**Table 1:** Location coordinates and weather parameter of the studied cumin landraces.

Location name	Location coordinates	Elevation (MSL)	Mean annual rainfall
North Khorasan	37°09′42′′N, 57°03′20.30′′E	1356.6	130 mm
Golestan	37°14′17.9′′N, 55°09′34.8′′E	37.5	363 mm

**Table 2:** The different combination of PGRs (2,4-D and Kin) used in the present study.

Medium code	2,4-D (mg/L)	Kin (mg/L)
A	0.5	0.1
B	1	0.1
C	2	0.1
D	0.5	—
E	1	—
F	2	—
G	—	0.1

**Table 3:** Chemical composition of the essential oil of two studied Iranian superior cumin landraces.

S. number	Compounds%	RI	Golestan landrace	RI	North Khorasan landrace
*Terpenic compound*					
1	Alpha-thujene	1453	17.22 ± 0.26^^b^^	1090	24.8 ± 0.10^a^
2	Gamma-terpinene	998	13.35 ± 0.21^a^	998	6.91 ± 0.11^b^
3	Alpha-pinene	948	0.45 ± 0.005^a^	948	0.14 ± 0.017^b^
4	Alpha-phellandrene	969	0.76 ± 0.01^a^	969	0.73 ± 0.005^a^
5	Beta-farnesene	1440	0.58 ± 0.01^a^	—	—
6	Beta-pinene	943	9 ± 0.22^a^	943	3.35 ± 0.09^b^
7	Beta-phellandrene	964	0.50 ± 0.01^a^	964	0.27 ± 0.02^b^
8	Bergamotene	1425	0.12 ± 0.005^a^	—	—
9	Di-epi-.alpha.-cedrene	1403	0.65 ± 0.017^a^	1403	0.18 ± 0.008^b^
10	Myrcene (b-pinene)	—	—	958	0.26 ± 0.005^a^
11	Limonene	—	—	1013	0.42 ± 0.008^a^
12	Beta-myrcene	958	0.56 ± 0.03^a^	—	—
13	B-Cymene	1042	5.48 ± 0.08^a^	1042	2.58 ± 0.03^b^
14	Gamma-cadinene	1435	0.42 ± 0.01^a^	—	—
15	*β*-Bisabolene	1500	0.10 ± 0.003^a^	—	—
16	Beta-caryophyllene	1494	0.25 ± 0.003^a^	—	—
17	Cyclopropane	928	0.52 ± 0.04^a^	—	—
18	Androstane	—	—	2145	0.07 ± 0.003^a^
19	Origanene	902	0.14 ± 0.008^a^	902	0.06 ± 0.006^b^
20	1,5-Cyclodecadiene	1570	0.08 ± 0.00^a^	—	—
21	Beta-caryophyllene oxide	1507	0.06 ± 0.003^a^	—	—
22	Cholestane	—	—	2770	0.23 ± 0.008^a^
	3-Nonen-5-yne, 4-ethyl-, (Z)	1117	11.22 ± 0.20^a^	1117	11.14 ± 0.08^a^
23	Benzene –Dimethoxymethyl	1080	0.27 ± 0.01^a^	—	—
24	Silane	1115	0.56 ± 0.008^a^	—	—

Total terpenic compound			**62.27**		**51.14**

*Alcohols/phenols*	p-Thymol	1262	0.14 ± 0.003^a^	—	—
	Isoeugenol	—	—	1410	1.18 ± 0.03^a^
	Phenol, 3-(1-methylethyl)	1149	0.05 ± 0.005^b^	1149	0.15 ± 0.006^a^
	p-Cymen-8-ol	1197	0.07 ± 0.003^a^	—	—
	Carotol	1593	0.35 ± 0.008^a^	1593	0.06 ± 0.006^b^
	*cis*-Sabinol	1085	0.07 ± 0.003^a^	1085	0.06 ± 0.0^a^
	*β*-linalool	1082	0.13 ± 0.005^b^	1082	0.24 ± 0.006^a^
	p-Mentha-1,4-dien-7-al	1240	0.12 ± 0.003^b^	1240	0.44 ± 0.031^a^
	Phellandral	1175	4.38 ± 0.12^b^	1175	6.91 ± 0.08^a^
	*trans*-Pinocarveol	1131	0.11 ± 0.003^b^	1131	0.14 ± 0.006^a^
	1,8-Cineole	1247	0.23 ± 0.005^a^	—	—
	Henicosan-1-ol	2351	0.09 ± 0.001^a^	—	—
	*trans*-Shisool	—	—	1250	0.20 ± 0.005^a^
	Terpinen-4-ol	1137	0.14 ± 0.003^b^	1137	0.23 ± 0.01^a^

Total alcohols/phenols			**5.88**		**9.61**

*Aldehydes*	Cuminaldehyde	1230	30.4 ± 0.27^b^	1230	37.8 ± 0.24^a^
	Carene	919	0.09 ± 0.001^a^	919	0.078 ± 0.0016^b^
	Nonanal	1104	0.43 ± 0.008^a^	1104	0.32 ± 0.005^b^
	Cyclopentene-1 carboxaldehyde	—	—	1176	0.20 ± 0.006^a^

Total aldehydes			**30.91**		**38.4**

*Epoxides*	Limonene epoxide	1031	0.05 ± 0.003^a^	—	—

Total epoxides			**0.05**		**0**

*Others*	Dehydro-neotigogenin benzoate	—	—	4336	0.70 ± 0.01^a^
	Corylon	972	0.12 ± 0.006^a^	—	—

—: not detected. In each row, values followed by the same letter indicate no significant differences at 5% (*T* test). Means of five replications ± SE. RI: retention index determined by gas chromatography-mass spectrometry (GC-MS).

**Table 4:** Mean comparisons of medium × landrace × explant effect in the induction of callus production and related characters in Iranian superior cumin landraces.

Medium code	Landrace	Explant	% callus production	Callus area (cm)	Callus perimeter (cm)
A	Golestan	Cotyledon	93.4 ± 6.8^a^	0.93 ± 0.09^cd^	4.99 ± 0.16^bc^
A	Golestan	Hypocotyl	93.4 ± 6.8^a^	1.13 ± 0.12^bc^	4.91 ± 0.34^bc^
A	North Khorasan	Cotyledon	86.7 ± 13.7^ab^	1.63 ± 0.07^a^	6.00 ± 0.37^a^
A	North Khorasan	Hypocotyl	86.7 ± 6.8^ab^	1.01 ± 0.15^bcd^	4.53 ± 0.31^bcde^
B	Golestan	Cotyledon	100 ± 0^a^	1.24 ± 0.24^b^	5.02 ± 0.42^b^
B	Golestan	Hypocotyl	93.4 ± 6.8^a^	0.86 ± 0.2^d^	3.86 ± 0.27^efgh^
B	North Khorasan	Cotyledon	93.4 ± 6.8^a^	0.79 ± 0.04^de^	4.17 ± 0.04^defg^
B	North Khorasan	Hypocotyl	86.7 ± 6.8^ab^	0.83 ± 0.17^de^	3.99 ± 0.43^efgh^
C	Golestan	Cotyledon	100 ± 0^a^	0.87 ± 0.01^d^	4.71 ± 0.17^bcd^
C	Golestan	Hypocotyl	86.7 ± 6.8^ab^	0.66 ± 0.0^e^	3.62 ± 0.05^fgh^
C	North Khorasan	Cotyledon	100 ± 0^a^	0.79 ± 0.09^de^	3.58 ± 0.03^fgh^
C	North Khorasan	Hypocotyl	80 ± 11.7^ab^	0.68 ± 0.01^e^	3.53 ± 0.006^gh^
D	Golestan	Cotyledon	100 ± 0^a^	0.80 ± 0.5^de^	4.08 ± 0.5^defg^
D	Golestan	Hypocotyl	80 ± 11.7^ab^	0.58 ± 0.06	3.37 ± 0.19^h^
D	North Khorasan	Cotyledon	93.4 ± 6.8^a^	0.84 ± 0.02^de^	3.71 ± 0.07^efgh^
D	North Khorasan	Hypocotyl	86.7 ± 6.8^ab^	0.73 ± 0.03^e^	3.58 ± 0.04^fgh^
E	Golestan	Cotyledon	93.4 ± 6.8^a^	0.72 ± 0.04^e^	3.67 ± 0.08^efgh^
E	Golestan	Hypocotyl	80 ± 11.7^ab^	0.61 ± 0.04^e^	3.36 ± 0.12^h^
E	North Khorasan	Cotyledon	93.4 ± 6.8^a^	0.73 ± 0.03^e^	3.59 ± 0.07^fgh^
E	North Khorasan	Hypocotyl	66.7 ± 18^b^	0.63 ± 0.04^e^	3.38 ± 0.13^h^
F	Golestan	Cotyledon	100 ± 0^a^	0.98 ± 0.15^cd^	4.33 ± 0.50^cde^
F	Golestan	Hypocotyl	66.7 ± 18^b^	0.77 ± 0.02^de^	3.53 ± 0.12^gh^
F	North Khorasan	Cotyledon	100 ± 0^a^	0.82 ± 0.02^de^	4.02 ± 0.2^efgh^
F	North Khorasan	Hypocotyl	93.4 ± 6.8^a^	0.83 ± 0.3^de^	3.74 ± 0.1^efgh^
G	Golestan	Cotyledon	94.4 ± 5.77^a^	0.90 ± 0.03^cd^	4.22 ± 0.11^def^
G	Golestan	Hypocotyl	86.7 ± 6.8^ab^	0.98 ± 0.05^cd^	4.31 ± 0.21^de^
G	North Khorasan	Cotyledon	100 ± 0^a^	0.87 ± 0.03^d^	4.01 ± 0.09^defgh^
G	North Khorasan	Hypocotyl	80 ± 11.7^ab^	0.96 ± 0.05^cd^	4.13 ± 0.15^defg^

Mean values in each column followed by different letters indicate significant differences according to the DMRT test at *P* < 0.05.

**Table 5:** Mean comparisons of the effect of different mediums on the rooting and survival of regenerated plantlets in Golestan landrace.

Medium	Main rooting induction%	Days to initiation of main rooting induction%	Lateral rooting induction%	Ex vitro survival%
PGR-free	0.13 ± 0.05^c^	12.3 ± 0.88^a^	0.13 ± 0.03^b^	0^b^
IAA (0.35 mg/L)	0.27 ± 0.21^c^	11 ± 1.15^ab^	0.23 ± 0.13^b^	0.1 ± 0.03^b^
NAA (0.37 mg/L)	0.43 ± 0.15^b^	10.3 ± 0.9^ab^	0.27 ± 0.1^b^	0.17 ± 0.09^b^
IAA (0.35 mg/L) + NAA (0.37 mg/L)	0.67 ± 0.06^a^	9.33 ± 0.67^b^	0.63 ± 0.07^a^	0.58 ± 0.08^a^

Mean values in each column followed by different letters indicate significant differences according to the DMRT test at *P* < 0.05.

## Abbreviations

PGRs: Plant growth regulators
IAA: Indole-3-acetic acid
IBA: Indole-3-butyric acid
NAA: 1-Naphthaleneacetic acid
BAP: 6-Benzylaminopurine
2,4-D: 2,4-Dichlorophenoxyacetic acid
Kin: Kinetin
PEG: Polyethylene glycol
MS: Murashige and Skoog
B5: Gamborg's B5
CTAB: Cetyl trimethylammonium bromide
RAPD: Random Amplification of Polymorphic DNA
RFLP: Restriction Fragment Length Polymorphism
PCR: Polymerase chain reaction
GC-MS: Gas chromatography-mass spectrometry
ANOVA: Analysis of Variance
SE: Standard error
DMRT: Duncan's Multiple Range Test.
